# microRNA‐222 promotes colorectal cancer cell migration and invasion by targeting MST3

**DOI:** 10.1002/2211-5463.12623

**Published:** 2019-04-02

**Authors:** Fei Luo, Jianfeng Zhou, Shihua Wang, Zhao Sun, Qin Han, Chunmei Bai

**Affiliations:** ^1^ Department of Oncology Peking Union Medical College Hospital Chinese Academy of Medical Sciences Peking Union Medical College Beijing China; ^2^ Institute of Basic Medical Sciences Chinese Academy of Medical Sciences School of Basic Medicine Peking Union Medical College Beijing China

**Keywords:** colorectal cancer, invasion, migration, miR‐222, MST3

## Abstract

Metastasis is one of the major causes of death in colorectal cancer (CRC) patients. MiR‐222 has been reported to be an oncogene in many types of cancer. However, its role in CRC cell invasion and migration as well as CRC downstream signaling pathways remains largely unknown. Our study found that miR‐222 overexpression promotes the migration and invasion of CRC cell lines, and miR‐222 interference results, as expected, in inhibition of migration and invasion. Bioinformatic analysis and dual luciferase reporter assay showed that mammalian STE20‐like protein kinase 3 (*MST3*) may be the target gene of miR‐222. Down‐expression of MST3 in CRC cell lines enhanced their migration and invasion, but overexpression of MST3 could attenuate miR‐222 overexpression in the promotion of migration and invasion in colorectal cell lines. HCT116 cell lines overexpressing miR‐222 were transplanted into nude mice resulting in more lung metastases than in the control group. Further study found that MST3 may play a role in paxillin phosphorylation to reduce adhesion, or increase the invadopodia. These findings demonstrate that miR‐222 modulates *MST3* and therefore plays a critical role in regulating CRC cell migration and invasion. Thus, miR‐222 may be a novel therapeutic target for CRC.

**Abbreviations**
CRC: colorectal cancer
FN: fibronectin
GAPDH: glyceraldehyde 3‐phosphate dehydrogenase
MST3: mammalian STE20‐like protein kinase 3
UTR: untranslated region

Colorectal cancer (CRC) is the third most common malignancy in males and the second in females worldwide, with 1.2 million new patients being diagnosed each year 1, 2. Metastasis is one of the major causes of death in CRC patients. Approximately 50–60% of CRC patients develop distant metastases after surgery and their 5‐year survival rate is less than 12% 3. Therefore, a better understanding of the mechanisms that govern CRC metastasis will provide information on novel biomarkers for oncologists to screen in patients with high metastatic risk and provide patients with customized treatments. Additionally, it may suggest new therapeutic targets to reduce the incidence of distant metastasis.

Many signaling pathways, such as transforming growth factor‐β/Smad, Wnt/β‐catenin and mitogen‐activated protein kinase, have been reported to regulate CRC metastasis 4. In addition to signaling pathways, the roles of non‐coding RNAs in CRC metastasis have also been intensively investigated. MiRNAs, a class of small, non‐coding RNAs, can bind to the 3′‐untranslated region (UTR) of target genes and regulate gene expression at the post‐transcriptional level 5. Recently, a number of groups have demonstrated abnormal miRNA expression in CRC tissues compared to normal tissues 6, 7, 8, 9, 10, 11, 12, and significant progress has been made in elucidating the specific roles of miRNAs in the initiation, progression and metastasis of CRC. For example, as a highly expressed onco‐miRNA in many cancers, including CRC, high miR‐21 expression is correlated with chemotherapy insensitivity and poor prognosis 10. MiR‐143 and miR‐145 can exert tumor‐suppressive functions and have low expression in CRC 13. MiR‐543 inhibited CRC cell metastasis *in vitro* and *in vivo* by targeting *KRAS*,* MTA1* and *HMGA2* 14. MiR‐34a‐5p suppresses metastasis and its expression predicts recurrence in patients with stage II/III CRC 15. Pro‐metastatic effects of miRNAs in CRC have also been reported. For example, miR‐625‐3p exerts its oncogenic functions in CRC metastasis by regulating the suppressor of cancer cell invasion/E‐cadherin/matrix metalloproteinase‐9 pathways 16.

Increased levels of miR‐222 have been reported in plasma as well as in tumor tissues from CRC patients 17, 18. MiR‐222 can promote CRC cell proliferation by promoting a positive feedback loop to increase RelA and signal transducer and activator of transcription 3 expression levels 19. Stinson *et al*. 20 reported that miR‐221/222 promoted breast cancer migration and invasion by down‐regulating *TRPS1* expression, which suggested that *TRPS1* is a tumor suppressor gene in breast cancer. However, Hong *et al*. 21 reported that *TRPS1* was an oncogene in CRC. Therefore, the mechanism by which miR‐222 affects the migration and metastasis of CRC still needs further research. In this study, we found that overexpression of miR‐222 significantly promoted migration and invasion of CRC cells through MST3, whose expression is inversely correlated with miR‐222 in CRC specimens and predicted disease‐free survival. Our study suggests that miR‐222 may be a critical determinant of CRC metastasis.

## Materials and methods

### Clinical specimens

Twenty‐one CRC tissue samples were obtained from patients with CRC treated between 2014 and 2015 and were stored liquid nitrogen‐frozen.

The study conformed the guidelines set by the Declaration of Helsinki. The patients were enrolled after obtaining their informed consent according to procedures approved by the Ethics Committee at Peking Union Medical College hospital.

### Tumor cell culture

The human SW480, HCT8, HCT116 and Lovo CRC cell lines were purchased from Cell Resource Center, IBMS, CAMS/PUMC (Beijing, China). SW480 was established from a primary colon adenocarcinoma in a 50‐year‐old male. HCT8 was derived from an ileocecal colorectal adenocarcinoma in a 67‐year‐old male. HCT116 was derived from a colorectal carcinoma in a male adult. Lovo was derived from a colorectal adenocarcinoma in a 56‐year‐old male patient. All of the cell lines were maintained in Dulbecco's modified Eagle's medium (Thermo Fisher Scientific, Waltham, MA, USA) supplemented with 10% fetal calf serum (Thermo Fisher Scientific) in a humidified 5% CO_2_ atmosphere at 37 °C.

### miRNA and siRNA transfection

miRNA and siRNA transfections were performed as previously described 22. The synthetic miR‐222 mimic (forward, 5′‐AGC UAC AUC UGG CUA CUG GGU‐3′ and reverse, 5′‐CCA GUA GCC AGA UGU AGC UUU‐3′), miR‐222 inhibitor (5′‐ACC CAG UAG CCA GAU GUA GCU‐3′), mimic control (forward, 5′‐UUC UCC GAA CGU GUC ACG UTT‐3′ and reverse, 5′‐ACG UGA CAC GUU CGG AGA ATT‐3′) and inhibitor control (5′‐CAG UAC UUU UGU GUA GUA CAA‐3′) were purchased from GenePharma, Inc., Shanghai, China, and have been previously described 23. The si‐MST3 sequence is 5′‐GGA GAA GAG CCA GGC GTG C‐3′ 24.

### RNA reverse transcription and qRT‐PCR

Total RNA was extracted using the TRIzol total RNA isolation reagent (Thermo Fisher Scientific) and purified with the Column DNA Erasol kit (Tiangen, Beijing, China) according to the manufacturers’ instructions. In the reverse transcription step, cDNAs were synthesized using a high‐capacity cDNA reverse transcription Kit (Thermo Fisher Scientific) and levels were assessed with qRT‐PCR using SYBR Green I (TaKaRa, Dalian, China). The gene expression levels were normalized to the endogenous reference gene *GAPDH*. The experiments were performed in triplicate. Reverse transcription of miRNAs was performed with a miScript Reverse Transcription Kit (Hilden, Duesseldorf, Germany). The expression of mature miRNAs was determined using miRNA‐specific qRT‐PCR (TaKaRa). The primers are listed in Tables S1 and S2.

### Western blot analysis

This protocol was performed as previously described 25. After washing twice with PBS, the cells were lysed in ice‐cold Radio Immunoprecipitation Assay lysis buffer (Beyotime, Nanjing, China) and manually scraped from the culture plates. Proteins were separated on 10% SDS/PAGE gels, electroblotted onto a polyvinylidenedifluoride membrane and incubated with anti‐MST3 antibody (1/1000; cat. no. 3723S; Cell Signaling Technology, Danvers, MA, USA) and anti‐glyceraldehyde 3‐phosphate dehydrogenase (GAPDH) antibody (1/1000; cat. no. sc‐20358; Santa Cruz Biotechnology, Dallas, TX, USA), followed by incubation with a secondary anti‐rabbit or anti‐mouse horseradish peroxidase‐conjugated antibody (1/3000; Santa Cruz Biotechnology). Antibody‐antigen complexes were detected using a chemiluminescent ECL reagent (Millipore Corp., Billerica, MA, USA).

### *In vitro* migration and invasion assay

Transwell chambers (8 μm pore size; Costar, Kennebunk, ME, USA) were used in the *in vitro* migration and invasion assay. For migration assays, CRC cells or the same number of corresponding miR‐222‐overexpressing or miR‐222‐ knockdown cells were seeded in the top chambers. For invasion assays, CRC cells or the same number of corresponding miR‐222‐overexpressing or miR‐222‐knockdown cells were seeded in the top chamber membranes, which were coated with Matrigel. The migrating or invading cells were counted and photographed. Because SW480, HCT8, HCT116 and Lovo cells have different migration and invasion capacities, transwell assays differed slightly among them. For the SW480 cells, the cell seeding number was 4 × 10^5^ cells and the time for the migration and invasion assays was 24 and 72 h, respectively. For the HCT8 cell, the cell seeding number was 2 × 10^5^ and the time for the migration and invasion assays was 12 and 36 h, respectively. For the HCT116 and Lovo cells, the cell seeding number was 1 × 10^5^ and the time for the migration and invasion assays was 12 and 36 h, respectively.

### Dual luciferase reporter gene construct and dual luciferase reporter assay

To identify potential miR‐222 targets, we first used TargetScan and PicTar web to predict targets. Based on known miRNAs, mRNAs that may be targeted are predicted, and genes associated with tumor invasion and invasion were selected based on the function of the mRNA. Fragments from the MST3, PTEN, TIMP2, RECK and PLXNC1 3′‐UTRs contained the predicted binding site for hsa‐miR‐222, and flanking sequences on each side were synthesized with a short extension containing cleavage sites for XhoI (5′‐end) and NotI (3′‐end) (Table S3); a second fragment containing a mutated binding site sequence was also synthesized. The two constructs were termed wild‐type (WT) and mutant (MT). The fragments were cloned into the psiCHECK™‐2 vector (Promega Corp., Madison, WI, USA). Ten nanograms of WT, MT or control vector and 200 nm miR‐222 mimic were transfected into 293T cells using Lipofectamine 2000 (Thermo Fisher Scientific) according to the manufacturer's instructions. The cells were harvested 24 h after transfection and assayed for *Renilla* and firefly luciferase activities using the Dual Luciferase Reporter Assay System (Promega Corp.). This protocol was performed as previously described 23, 26.

### MST3 overexpression

A plasmid vector of the MST3 pReceiver‐M68 expression clone was purchased from FulenGene (EX‐T8396‐M68, Guangzhou, China). MST3 overexpression or control plasmid was transfected into CRC cell lines with Lipofectamine 3000 (Thermo Fisher Scientific). The transfection efficacy was determined by RT‐PCR at 24 h and by western blot at 48 h.

### Immunofluorescence experiments

Cells were fixed at room temperature in 4% paraformaldehyde for 10 min, permeabilized with cold acetone for 10 min, and stained with MST3 antibody (1 : 100, Cell Signaling Technology) at room temperature for 1 h. After washing with PBS, a second antibody was added (Alexa Fluor® 488‐conjugated anti‐rabbit IgG, 1 : 200; Abcam, Cambridge, MA, USA); after washing, cells were incubated with 0.1 μg·mL^−1^ Hoechst 33342 (Thermo Fisher Scientific), before being observed under a confocal microscope (Olympus, Tokyo, Japan).

### Invadopodia assay

To detect invadopodia, cells were fixed at room temperature in 4% paraformaldehyde for 10 min and permeabilized with cold acetone for 10 min. Next,cells were stained with rhodamine‐conjugated phalloidin (1 : 200; Thermo Fisher Scientific) and a Alexa Fluor® 488‐conjugated anti‐cortactin antibody (1 : 200; Abcam) at room temperature for 1 h; after washing, cells were incubated with 0.1 μg·mL^−1^ Hoechst 33342, before being observed under a confocal microscope (Olympus).

### Adhesion assay

According to the reported method of adhesion assay, 100 μg·mL^−1^ fibronectin (FN) was added to 96‐well culture plates at 100 μL per well 27. Plates were incubated overnight at 4 °C. After washing once with PBS, it was blocked with 1% BSA for 2 h at 37 °C. After washing twice with RPMI 1640 medium, the coated cell culture plates were used for an adhesion assay; the BSA control group was not coated with FN, and treated with 1% BSA alone. Cells at 0.5 × 10 ^6^ cells per mL were added to the FN‐coated cell culture plate and 1% BSA blocked culture plate at 100 μL per well, and the plates incubated in a CO_2_ incubator for 60 min at 37 °C. Non‐adherent cells were aspirated, plates were washed twice with PBS, and attached cells were stained with crystal violet (dissolved in glacial acetic acid), and detect with a plate reader at 570 nm. The number of adherent cells (*D*) was the experimental group *D* minus the BSA control group.

### Lentiviral transduction particle preparation and transduction

This protocol was performed as previously described 25. MiR‐222 and a negative control expression cassette were first constructed in the pGLV10/U6/RFP/Puro vector (GenePharma). The inserted hsa‐mir‐222 sequence was 5′‐GCTGCTGGAAGGTGTAGGTACCCTCAATGGCTCAGTAGCCAGTGTAGATCCTGTCTTTCGTAATCAGCAGCTACATCTGGCTACTGGGTCTCTGATGGCATCTTCTAGCT‐3′. Lentiviral transduction particles were prepared and provided by GenePharma, Inc. Successfully transduced HCT116 cells were selected in culture medium containing puromycin (2 μg·mL^−1^) 24 h after transduction. The transduction efficiency was evaluated by detecting red fluorescent protein expression under a fluorescence microscope, and the miR‐222 expression level was determined by qRT‐PCR 2 weeks after selection.

### Evaluation of metastasis regulation by miR‐222 in mice

This protocol was performed as previously described 25. Six‐ to eight‐week‐old male nude mice (BALB/c‐nu) were purchased from the Experimental Animal Institute of the Chinese Academy of Medical Sciences (Beijing, China). The mice were bred and maintained under specifically pathogen‐free conditions in individually ventilated (high‐efficiency particle‐arresting filtered air) sterile microisolator cages (Techniplast, Milan, Italy). All of the animal handling and experimental procedures were approved by the Animal Care and Use Committee of the Chinese Academy of Medical Sciences. HCT116‐miR‐222 and HCT116‐NC cells were prepared (5 × 10^6^ cells in 200 μL PBS) and inoculated subcutaneously at the right forelimb armpit. Histological evaluation of tumor metastasis in the liver, lung and colorectum was performed on these two groups of mice (HCT116‐miR‐222 and HCT116‐NC) every 2 weeks.

### Immunohistochemistry and assessment

Immunohistochemical staining was performed on 4 mm sections from 38 paraffin‐embedded specimens. The slides were baked at 65 °C for 30 min, deparaffinized with xylene and rehydrated with ethanol. For antigen retrieval, the slides were microwave‐treated and boiled in a 0.01 m citrate buffer (pH 6.0) for 10 min at 95 °C. Endogenous peroxidase activity was blocked with 3% hydrogen peroxide for 10 min. The slides were incubated with anti‐MST3 antibody (1/100; cat number 3723S; Cell Signaling Technology) overnight at 4 °C in a humidified chamber. The tissue sections were treated with biotinylated anti‐rabbit secondary antibody (Thermo Fisher Scientific), followed by further incubation with a streptavidin–horseradish peroxidase complex (Thermo Fisher Scientific). The antigen–antibody complexes were visualized using 3,3′‐diaminobenzidine, counterstained with 10% Mayer's hematoxylin, dehydrated, and mounted in Crystal Mount. The degree of immunostaining on the formalin‐fixed, paraffin‐embedded sections was reviewed and scored independently by two observers based on the proportion of positively stained cells and the intensity of the staining. If > 10% of the tumor cells or normal colon epithelial cells were stained, we defined the specimen as positive.

### Statistical analysis

Comparisons between groups were analyzed using Student's *t*‐test (two‐sided). Differences with *P* values of less than 0.05 are considered significant. Correlations between miR‐222 and MST3 expression were determined with Pearson's assay and correlation with the bivariate Kendall's tau‐b assay.

## Results

### miR‐222 promotes CRC cell line migration and invasion *in vitro*

**Figure 1:**
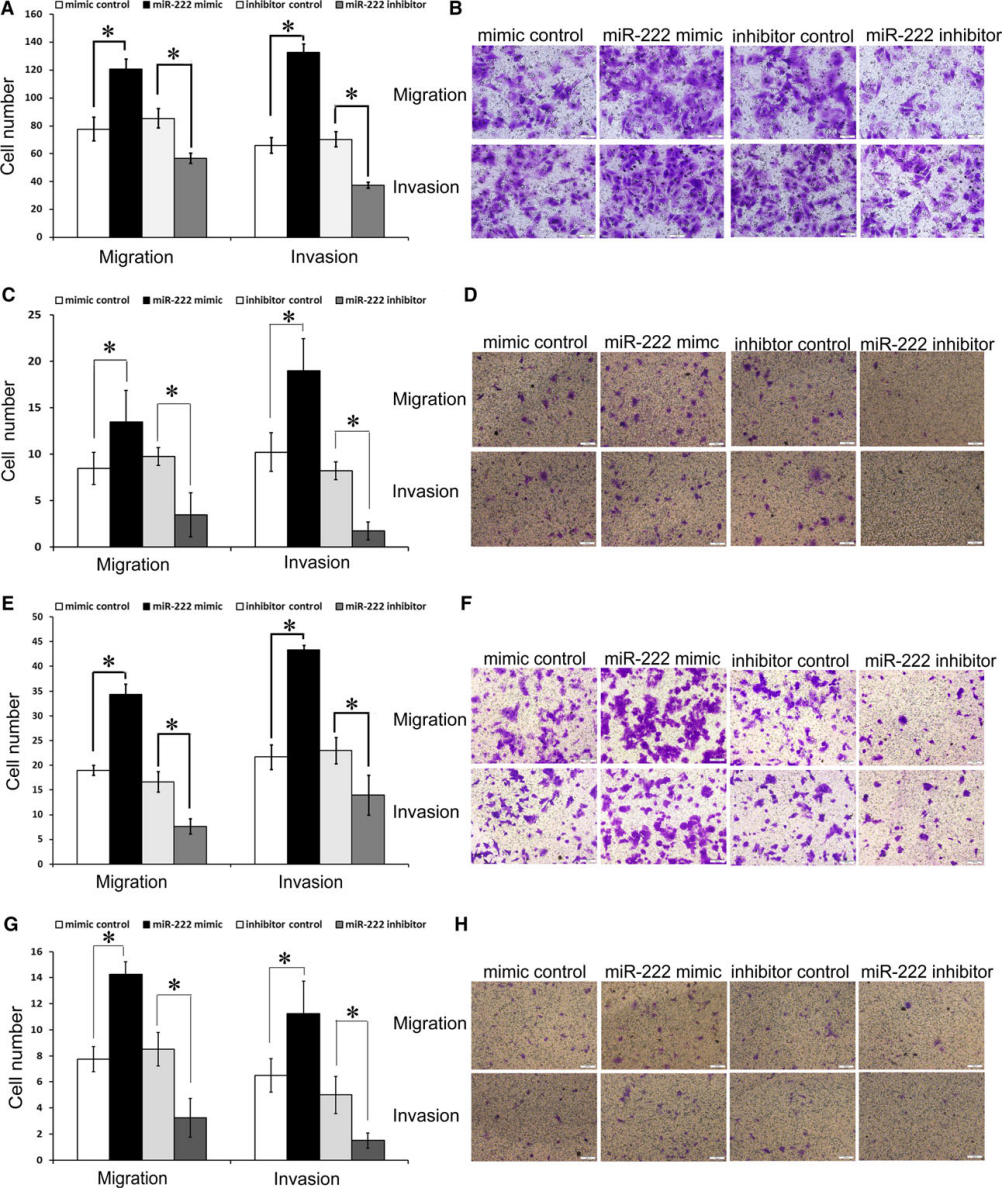
Effects of miR‐222 on migration and invasion in the SW480, HCT8, HCT116 and Lovo CRC cell lines. Transwell migration and invasion assays showed that CRC cells transfected with miR‐222 mimics had higher invasive and migratory capacities than the control cells (mimics control), while CRC cells transfected with the miR‐222 inhibitors had reduced invasive and migratory capacities compared to the control cells (inhibitor control) in SW480 (A,B), HCT8 (C,D), HCT116 (E,F) and Lovo cells (G,H). Statistical results (A,C,E,G) and microscopic images of crystal violet staining (B,D,F,H) are shown. Data represent the mean ± SD of four independent experiments. Comparisons between groups were analyzed using *t*‐tests (two‐sided). Differences with *P* values of less than 0.05 are considered significant. *P < 0.05. Scale bar: 100 μm (B,D,G,H).

To investigate the significance of miR‐222 in CRC, we first detected the expression of miR‐222 in the human SW480, HCT8, HCT116 and Lovo CRC cell lines by RT‐PCR. The Δ*C*_T_ value was used to represent the expression level of miR‐222 in each cell type. A higher ∆*C*_T_ indicated lower miR‐222 levels. As shown in Fig. S1, miR‐222 levels were higher in HCT116 and LOVO cells compared to SW480 and HCT8 cells. The human SW480, HCT8, HCT116 and Lovo CRC cell lines were then transfected with the miR‐222 mimic, mimic control, miR‐222 inhibitor and inhibitor control. The transfection efficiency is shown in Fig. S2. The four cell lines were next analyzed for their invasion and migration capacities via transwell chamber assays. Compared with the control group, CRC cells transfected with miR‐222 mimic had significantly higher invasion and migration capacities, while CRC cells transfected with the miR‐222 inhibitor had dramatically lower invasion and migration capacities (Fig. 1). These results suggested that miR‐222 promoted CRC invasion and migration.

### MiR‐222 directly decreases MST3 expression by interacting with its 3′‐UTR

**Figure 2:**
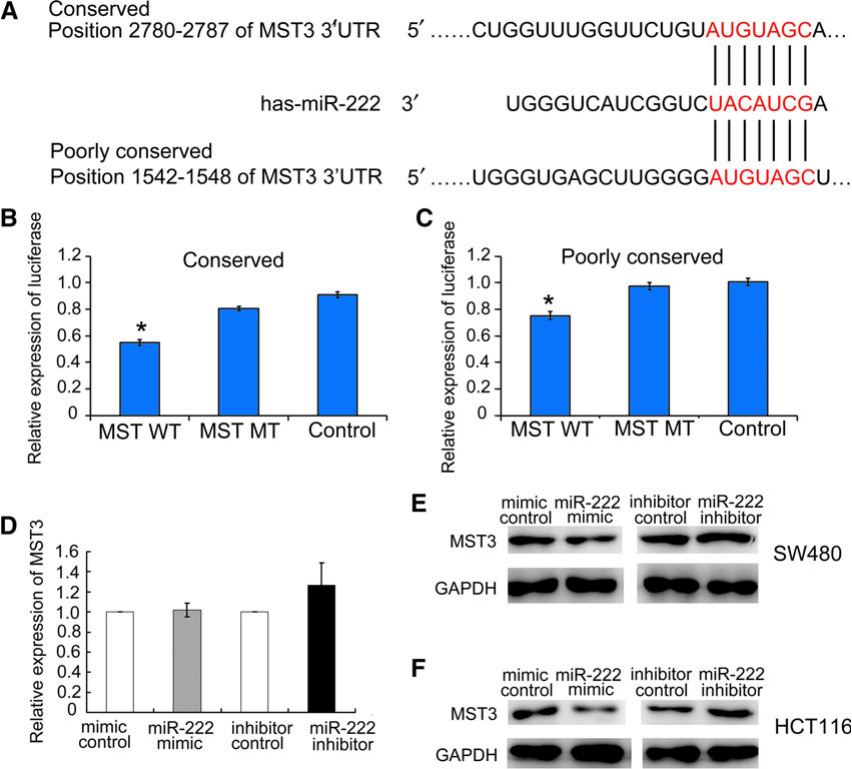
miR‐222 regulated MST3 by binding to its 3′‐UTR. (A) Putative miR‐222 binding sites in the MST3 3′‐UTR were revealed by TargetScan. (B,C) The luciferase activity after transfection of the indicated 3′‐UTR‐ or mutant 3′‐UTR‐driven reporter constructs. (B) Mutations in the conserved miR‐222 3′‐UTR binding site. (C) Mutations in the poorly conserved miR‐222 3′‐UTR binding site. Data represent the mean ± SD of at least three independent experiments. *P < 0.01. (D) RT‐PCR assay on MST3 mRNA levels in SW480 cells treated with miR‐222 mimics, mimics control, miR‐222 inhibitor and inhibitor control (*n* = 3). No statistical significance with *P* > 0.05. (E) Western blot assay of MST3 protein levels in SW480 cells treated with miR‐222 mimics, mimics control, miR‐222 inhibitor and inhibitor control (*n* = 3). (F) Western blot assay of MST3 protein levels in HCT116 cells treated with miR‐222 mimics, mimics control, miR‐222 inhibitor and inhibitor control (*n* = 3). Comparisons between groups were analyzed using *t*‐tests (two‐sided). Differences with *P* values of less than 0.05 are considered significant.

miRNAs exert their effects by regulating target genes at the post‐transcriptional level. To identify potential targets of miR‐222, we first used TargetScan and PicTar web to predict targets. Five genes, *MST3*,* PTEN*,* PLXNC1*,* RECK* and *TIMP2*, were selected based on the literature and their roles in migration and invasion. We next searched the literature for reported functions of these genes. The dual luciferase reporter assays revealed that the luciferase activities of MST3‐WT‐transfected cells significantly decreased upon miR‐222 overexpression. However, the inhibitory effects were abolished when the putative miR‐222 seed binding regions in the MST3 3′‐UTR were mutated (Fig. 2B,C). MiR‐222 overexpression had no effect on the luciferase activities of the other four genes (*PTEN*,* PLXNC1*,* RECK* and *TIMP2*; Fig. S3). Figure 2A demonstrates the conserved and poorly conserved miR‐222 binding sites in the MST3 3′‐UTR. MiR‐222 overexpression or down‐regulation had no effects on MST3 mRNA levels (Fig. 2D). MiR‐222 overexpression in SW480 cells and HCT116 cells reduced MST3 protein levels, while miR‐222 inhibition increased MST3 levels determined by western blot analysis (Fig. 2E,F). Similar results were obtained in HCT8 and Lovo cells (Fig. S4). Immunofluorescence staining of MST3 with miR‐222 overexpression or inhibition in four CRC cell lines showed the same trend of MST3 expression (Fig. S5). These data demonstrate that *MST3* is a direct target of miR‐222.

### Down‐regulation of MST3 increases invasion and migration in CRC cell lines

**Figure 3:**
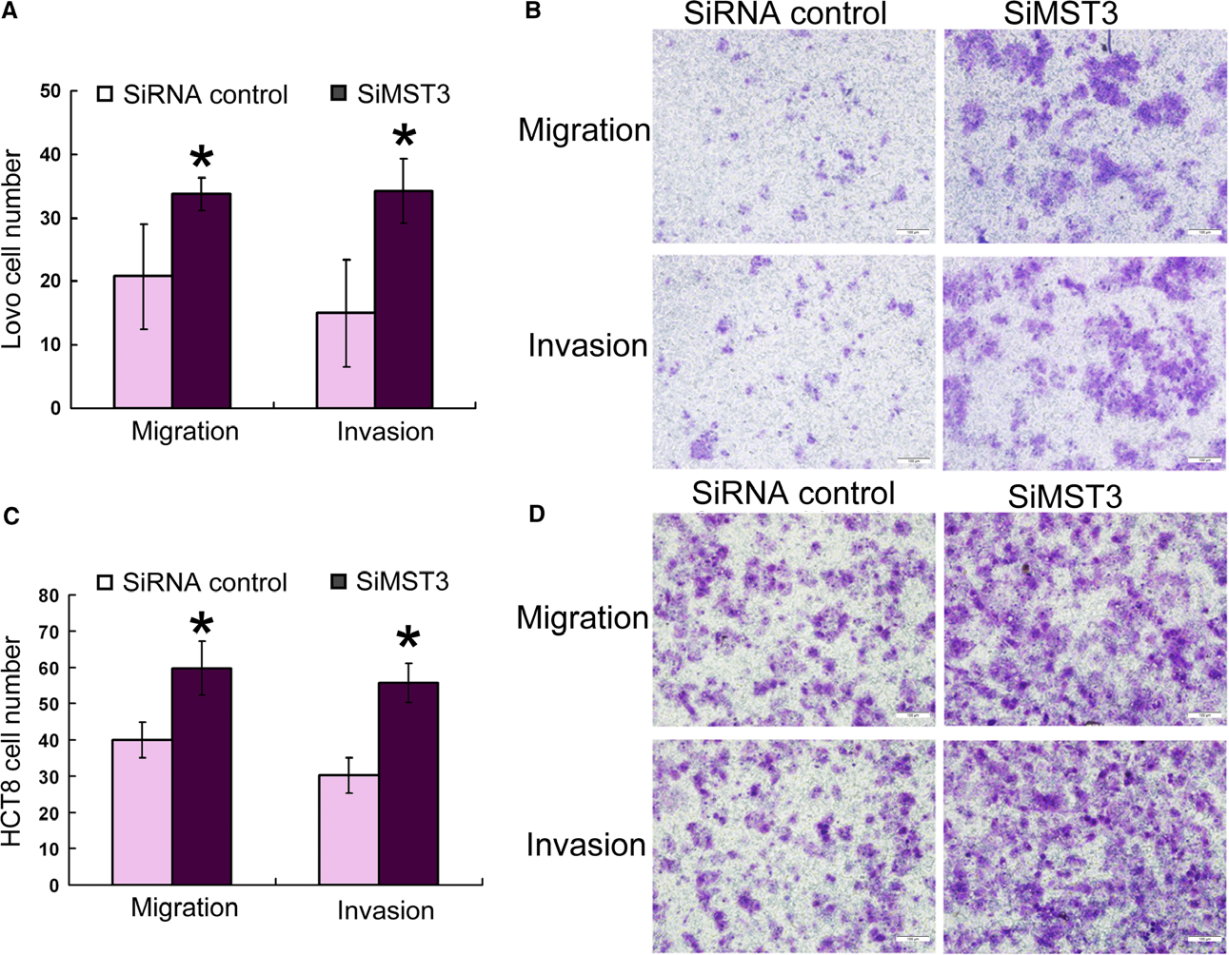
MST3 regulates HCT116 and HCT8 cell migration and invasion. Transwell migration (*n* = 4) and invasion (*n* = 4) assays showed that Lovo (A,B) and HCT8 (C,D) cells transfected with the MST3 siRNA had increased invasive and migratory capacities compared to the control cells (siRNA control). Statistical results (A,C) and microscopic images of Crystal violet staining (B,D) are shown. Data represent the mean ± SD of four independent experiments. *P < 0.01. Comparisons between groups were analyzed using *t*‐tests (two‐sided). Differences with *P* values of less than 0.05 are considered significant. Scale bar: 100 μm (B,D).

**Figure 4:**
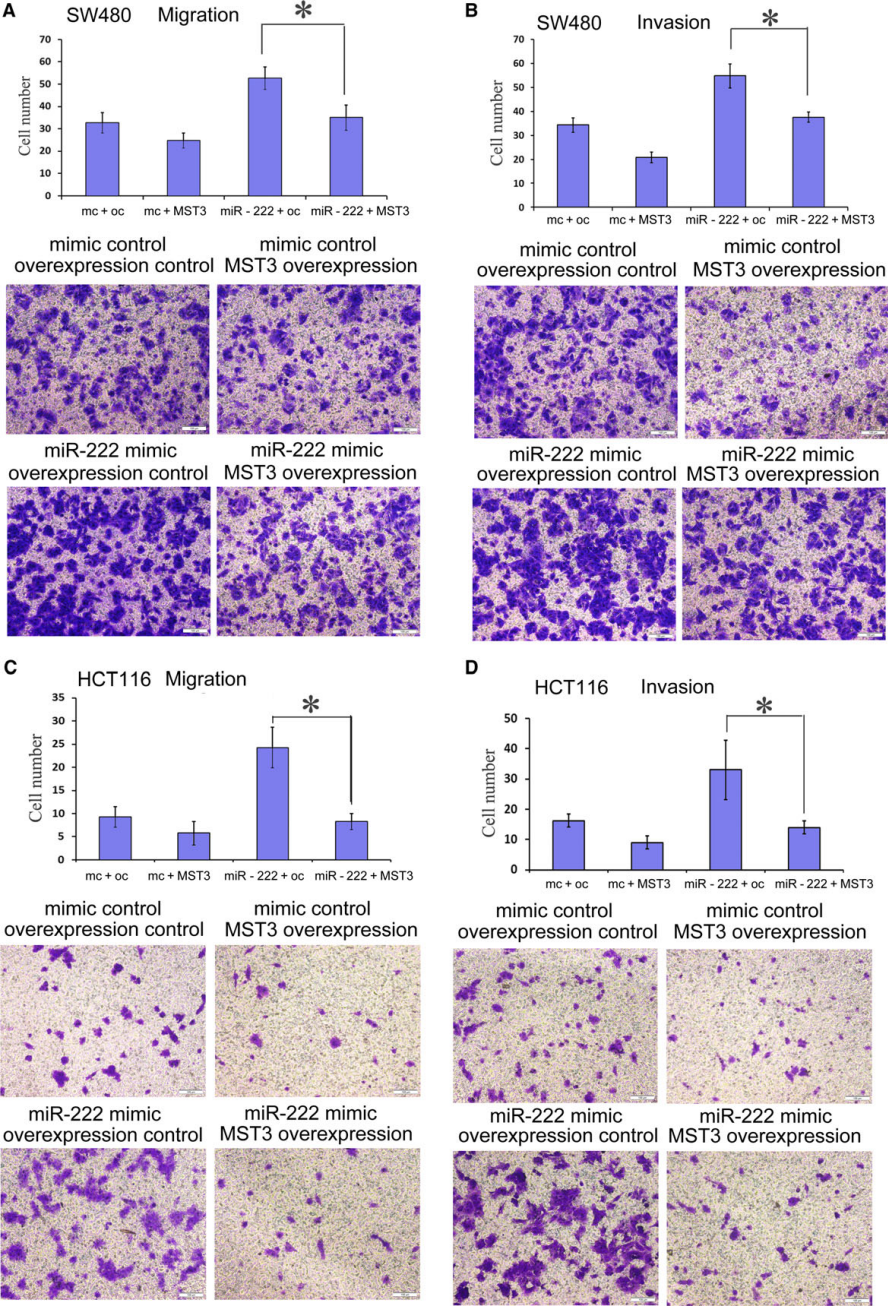
MST3 overexpression rescues the promoting effects of miR‐222 overexpression on CRC migration and invasion. Transwell migration (*n* = 4) (A,C) and invasion (*n* = 4) (B,D) assays showed that SW480 (A,B) and HCT116 (C,D) cells transfected with the MST3 expression vector had decreased invasive and migratory capacities compared to the control cells (control vector). Every lane shows the statistical results and microscopic images of Crystal violet staining. There were four groups: the mimic control and overexpression control group, mimic control and MST3 overexpression group, miR‐222 mimic and overexpression control group, and miR‐222 mimic and MST3 overexpression group. Data represent the mean ± SD of four independent experiments. Comparisons between groups were analyzed using *t*‐tests (two‐sided). Differences with *P* values of less than 0.05 are considered significant. *P < 0.01. Scale bar: 100 μm.

**Figure 5:**
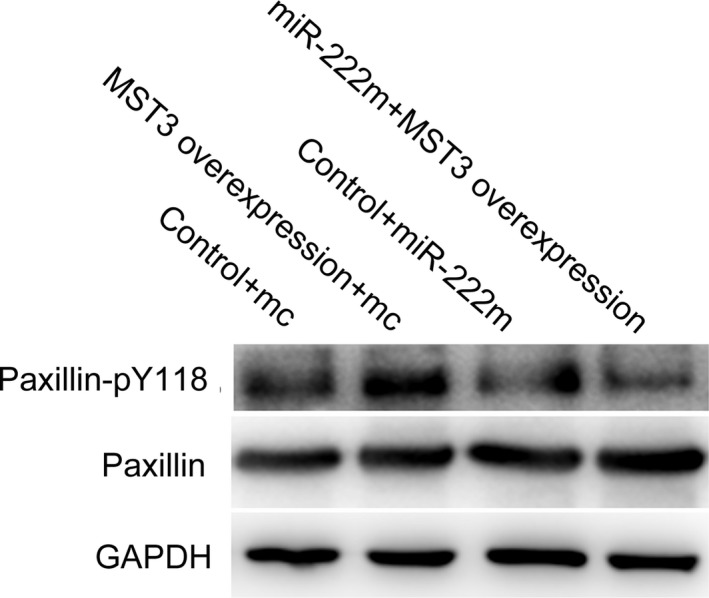
MST3 overexpression on paxillin phosphorylation. Western blot was performed to determine paxillin Y118 phosphorylation. Control + mimic represents the mimic control and overexpression control group, MST3 overexpression + mc represents the mimic control and MST3 overexpression group, control + miR‐222m represents the miR‐222 mimic and overexpression control group, and MST3 overpression + miR‐222mc represents the miR‐222 mimic and MST3 overexpression group.

The expression of MST3 in CRC cell lines was detected by western blot (Fig. S1B). To investigate whether MST3 down‐regulation could have effects on CRC cell migration and invasion, the expression of MST3 in HCT116 and HCT8 cells was suppressed by small interfering RNAs (siRNAs) (Fig. S6). The cell migration and invasion assay results showed that Lovo and HCT8 cell migration and invasion were significantly increased compared to the control group after MST3 down‐regulation (Fig. 3). We then evaluated the effect of MST3 overexpression. SW480 and HCT116 cells were transfected with MST3 overexpression or control plasmid vectors. The transfection efficiency is shown in Fig. S7. MST3 overexpression significantly reduced migration and invasion in SW480 and HCT116 cells (Fig. 4). To further confirm the miR‐222 effect was mediated by MST3, we performed an RNA interference rescue assay. Overexpression of MST3 could rescue the promoting effects of miR‐222 overexpression on CRC migration and invasion (Fig. 4). MST3 has been reported to regulate metastasis in breast cancer via paxillin, whose phosphorylation inhibits cell migration and invasion 24, 28. Here, we found increased paxillin phosphorylation in HCT8 cells transfected with the MST3 overexpression plasmid, and this effect was rescued when miR‐222 was also overexpressed (Fig. 5). These data suggest that miR‐222 affects CRC migration and invasion by targeting *MST3*, which subsequently regulates paxillin phosphorylation.

### miR‐222 and MST3 influence invadopodia formation and cell adhesion

**Figure 6:**
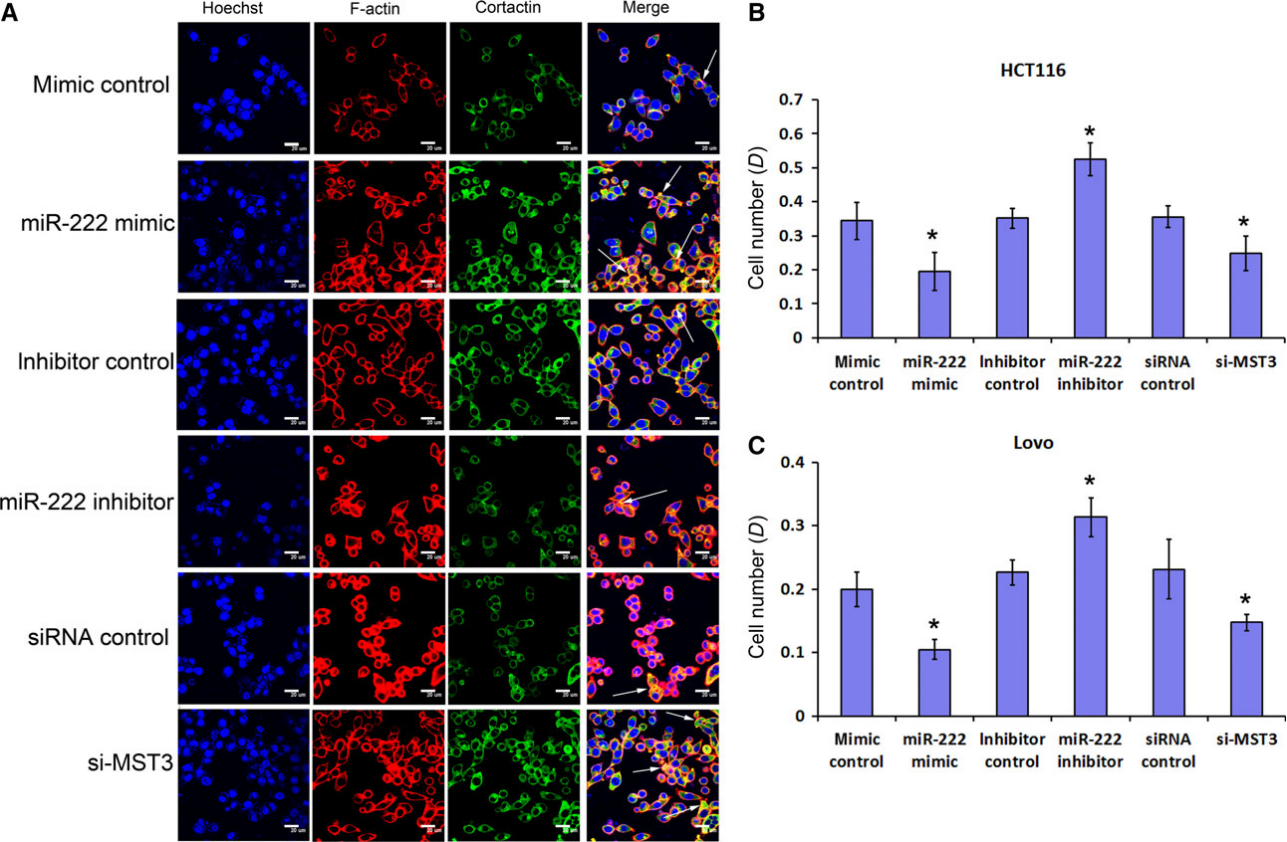
miR‐222 and MST3 influence HCT116 invadopodia formation and cell adhesion. (A) F‐actin (red) and cortactin (green) immunofluorescence images in HCT116 cells with miR‐222 overexpression (mimics), miR‐222 inhibitor, MST3 interference and the respective controls. F‐actin‐ and cortactin‐positive puncta are indicative of invadopodia. The nucleus is blue (stained by Hoechst 33342). Scale bar: 20 μm. (B,C) Adhesion assay in HCT116 cells (B) and Lovo cells (C) with miR‐222 overexpression (mimics), miR‐222 inhibitor, MST3 interference and the respective controls (*n* = 6). Data represent the mean ± SD of four independent experiments. Comparisons between groups were analyzed using *t*‐tests (two‐sided). Differences with *P* values of less than 0.05 are considered significant. *P < 0.01.

In order to further study the mechanism by which miR‐222 and MST3 regulate the migration and invasion of CRC cell lines, we investigated whether miR‐222 and MST3 affect the formation of invadopodia. After miR‐222 overexpression and MST3 interference, we found that invadopodia of HCT116 cells and Lovo cells were increased, and after miR‐222 interference, invadopodia were decreased (Figs 6A and S8).

In the previous study, we found that MST3 affects paxillin phosphorylation, whereas the main function of paxillin is focal adhesion. Therefore, we further investigated the effect of miR‐222 and MST3 on cell adhesion. The adhesion assay found that with miR‐222 overexpression and MST3 interference, CRC cell line adhesion was decreased, while with miR‐222 interference, CRC cell line adhesion was enhanced (Fig. 6B,C).

### miR‐222 promotes metastasis in mice

To further characterize the effects of miR‐222 in the CRC lines *in vivo*, we constructed HCT116 cells that stably overexpressed miR‐222 (HCT116‐miR‐222) and the respective control cells (HCT116‐NC) via lentiviral vector transfection. These cells were inoculated in the subcutaneous right forelimb armpit of nude mice. Mice from the two groups were sacrificed every 2 weeks to detect any developments of liver, lung or colorectal metastases. Until 6 weeks after inoculation, no metastases were detected in either group. At 8 weeks, lung metastatic masses were detected in the HCT116‐miR‐222 groups (4/5). In contrast, no metastases were detected after 8 weeks in the HCT116‐NC groups (0/5) (Fig. S9). No obvious liver or colorectal metastases were observed in either group.

### Correlation between MST3 and miR‐222 expression in CRC patients

The expression of MST3 and miR‐222 was detected by immunohistochemistry and RT‐PCR, respectively. The results showed that miR‐222 expression was inversely correlated to MST3 expression; these data were statistically significant (*P* = 0.034, Table S4). The 21 patients’ results are shown in Table S5.

## Discussion

miR‐222 has been reported to be overexpressed in many tumors and negatively correlated with prognosis, such as in non‐small cell lung cancer 29, gastric cancer 30 and bladder cancers 31. It can promote oncogenic processes such as cell proliferation, invasion and metastases by repressing a series of targets. For example, miR‐222 up‐regulation can enhance proliferation by down‐regulating its target P27 in lung cancer 32 and hepatocellular cancer 33. In oral squamous cell carcinoma, miR‐222 targeted the expression of p53 upregulated modulator of apoptosis and affected cell growth, invasiveness and apoptosis 34.

In CRC, Liu *et al*. 19 found that human CRC tissues had higher miR‐222 levels than non‐tumor colon tissues and that miR‐222 acted in a positive feedback loop to increase RelA and signal transducer and activator of transcription 3 expression levels. However, the role of miR‐222 in CRC cell invasion and migration as well as the underlying molecular mechanisms remains largely unknown. In this study, we found that miR‐222 overexpression significantly promoted CRC cell migration and invasion *in vitro* and *in vivo*. To understand the mechanism of miR‐222 actions in CRC, we predicted the potential target genes of miR‐222 via TargetScan and PicTar web. Five genes, *MST3*,* PTEN*,* PLXNC1*,* RECK* and *TIMP2*, were selected for further evaluation based on the prediction. Through dual luciferase reporter assays, we confirmed the direct targets of miR‐222 to specific sequences located in the MST3 3′‐UTR. *MST3* encodes a serine/threonine protein kinase that functions upstream of mitogen‐activated protein kinase signaling 35. MST3 is closely related to tumor initiation, proliferation and metastasis. In breast cancer, it is controversial whether *MST3* is an oncogene or a tumor suppressor gene. Lu *et al*. 24 found that *MST3* was a tumor suppressor gene and that its downregulation resulted in enhanced MCF7 migration. In contrast, Cho *et al*. 36 demonstrated that *MST3* was an oncogene that could promote proliferation and tumorigenicity in MDA‐MB‐231 cells. MST3 could regulate the mode of cancer cell migration by acting as the downstream molecule of the striatin‐interacting phosphatase and kinase complex 37. MiR‐222 directly targets the metastasis‐related protein MST3 24, 37, which can regulate paxillin phosphorylation 24. Paxillin is an intracellular adaptor protein that plays a key role in cytoskeletal organization. It can connect integrins to focal adhesion kinase and affect the assembly and disassembly of focal adhesions 38. We examined changes in the adhesion capacity of CRC cell lines after miR‐222 interference and overexpression as well as MST3 interference, and the results were as expected. Paxillin phosphorylation can inhibit cell migration and invasion 24, 28. Here, we found that MST3 could negatively regulate CRC cell invasion and migration. MST3 down‐regulation promoted whereas MST3 up‐regulation inhibited CRC cell migration and invasion. As a downstream target of MST3, we focused on paxillin, whose phosphorylation was increased by MST3. In addition, we also studied the effects of miR‐222 overexpression, interference and MST3 interference on invadopodia formation. We found that both miR‐222 overexpression and MST3 interference increased invadopodia formation in CRC cell lines. Therefore, miR‐222 and MST3 may regulate migration and invasion through the regulation of invadopodia formation. The mechanism needs further study. To our knowledge, this is the first study to report that *MST3* is a miR‐222 target gene and that miR‐222 can regulate CRC cell invasion and migration via MST3.

Furthermore, we also validated the *in vitro* results in clinical CRC patient tissues. MiR‐222 expression was inversely correlated with MST3 levels in CRC tissues.

In conclusion, high miR‐222 expression levels correlated with CRC migration and invasion. Our study showed for the first time that miR‐222 enhances CRC cell migration and invasion by down‐regulating MST3. The differential expression of MST3 between tumor and matched pericarcinous tissues may be useful for predicting prognosis in CRC patients.

## Conflict of interest

The authors declare no conflict of interest.

## Author contributions

All of the authors contributed to the concept and design of the study. FL and JZ collected and/or assembled data. SW, ZS, QH and CB analyzed and interpreted the data. All of the authors contributed to writing the manuscript. All of the authors approved the final manuscript.

## Supporting information


**Fig. S1**. MiR‐222 and MST3 expression in four cell lines. (A) miR‐222 expression in the SW480, HCT8, HCT116 and LOVO CRC cell lines as detected by RT‐PCR. (B) MST3 expression in the SW480, HCT8, HCT116 and LOVO CRC cell lines as detected by western blot.

**Fig. S2**. Transfection efficiency of miR‐222 mimics and inhibitors in SW480 cells as detected by RT‐PCR. Comparisons between groups were analyzed using *t*‐tests (two‐sided). Differences with *P* values of less than 0.05 are considered significant.

**Fig. S3**. The luciferase activity after transfection of the indicated 3′‐UTR‐ or mutant 3′‐UTR‐driven reporter constructs for PTEN, PLXNC1, RECK and TIMP2. Comparisons between groups were analyzed using *t*‐tests (two‐sided). Differences with *P* values of less than 0.05 are considered significant.

**Fig. S4**. Western blot assay of MST3 protein levels in HCT8 and Lovo cells treated with miR‐222 mimics, mimics control, miR‐222 inhibitor and inhibitor control (*n* = 3).

**Fig. S5**. Immunofluorescence assay of miR‐222 on MST3 expression in CRC cell lines. The nucleus is blue (stained by Hoechst 33342) and MST3 is green (stained by Alexa Fluor® 488). Scale bar: 20 μm.

**Fig. S6**. Interfering efficiency of MST3 siRNAs. (A,B) Interfering effects of MST3 siRNAs were detected by RT‐PCR. (A) HCT116 and (B) HCT8 cells (*n* = 3). Comparisons between groups were analyzed using *t*‐tests (two‐sided). Differences with *P* values of less than 0.05 are considered significant. (C) Western blot assay showed decreased MST3 expression after transfection with MST3‐siRNAs (200 nm).

**Fig. S7**. MST3 overexpression efficiency. MST3 expression was detected by RT‐PCR (A,C) (comparisons between groups were analyzed using *t*‐tests (two‐sided) and differences with *P* values of less than 0.05 are considered significant) and western blot (B,D) in SW480 (A,B) and HCT116 (C,D) cells.

**Fig. S8**. MiR‐222 and MST3 influence Lovo cell invadopodia formation. F‐actin (red) and cortactin (green) immunofluorescence images in Lovo cells with miR‐222 overexpression (mimics), miR‐222 inhibitor, MST3 interference and the respective controls. F‐actin‐ and cortactin‐positive puncta are indicative of invadopodia. The nucleus is blue (stained by Hoechst 33342). Scale bar: 20 μm.

**Fig. S9**. MiR‐222 promotes metastasis in mice. (A) Examination of lung tissues by hematoxylin‐eosin staining indicated no metastases in the HCT116‐NC inoculated mice (upper panel, 5/5) and metastases in the HCT116‐miR‐222 inoculated mice (lower panel, 4/5; the arrows indicate the metastatic sites). Scale bar: 100 μm. (B) Present overview picture of the lung sections in HCT116‐miR‐222 inoculated mice. Scale bar: 2 mm.

**Table S1**. The primers (mRNA) for real time PCR.
**Table S2**. The primers (miRNA) for real time PCR.
**Table S3**. The sequence of target gene wild‐type and mutation of 3′‐UTR.
**Table S4**. The correlation between miR‐222 and MST3 in 21 fresh liquid nitrogen‐frozen CRC cancer tissue from 2014 to 2015.
**Table S5**. The expression miR‐222 and MST3 of colorectal cancer patients (2014–2015).
