# Supplementary figures and images for: microRNA‐222 promotes colorectal cancer cell migration and invasion by targeting MST3
Source: FEBS Open Bio. 2019 Apr 2;9(5):901–13. doi: 10.1002/2211-5463.12623 (PMC6487838; doi:10.1002/2211-5463.12623)

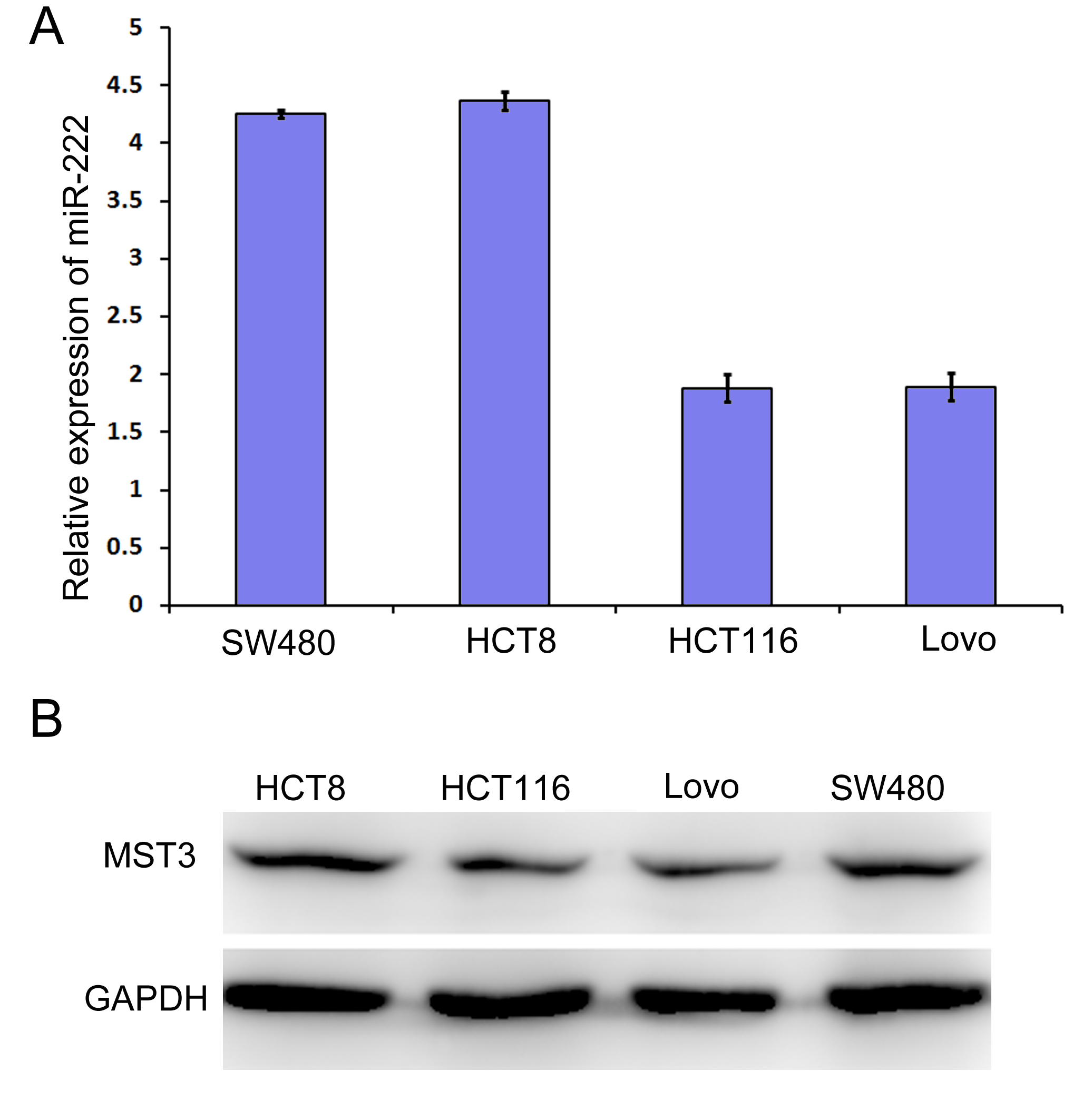

Supplement: Supplementary file 1 — Fig. S1. MiR‐222 and MST3 expression in four cell lines. (A) miR‐222 expression in the SW480, HCT8, HCT116 and LOVO CRC cell lines as detected by RT‐PCR. (B) MST3 expression in the SW480, HCT8, HCT116 and LOVO CRC cell lines as detected by western blot. [file FEB4-9-901-s001.tif]

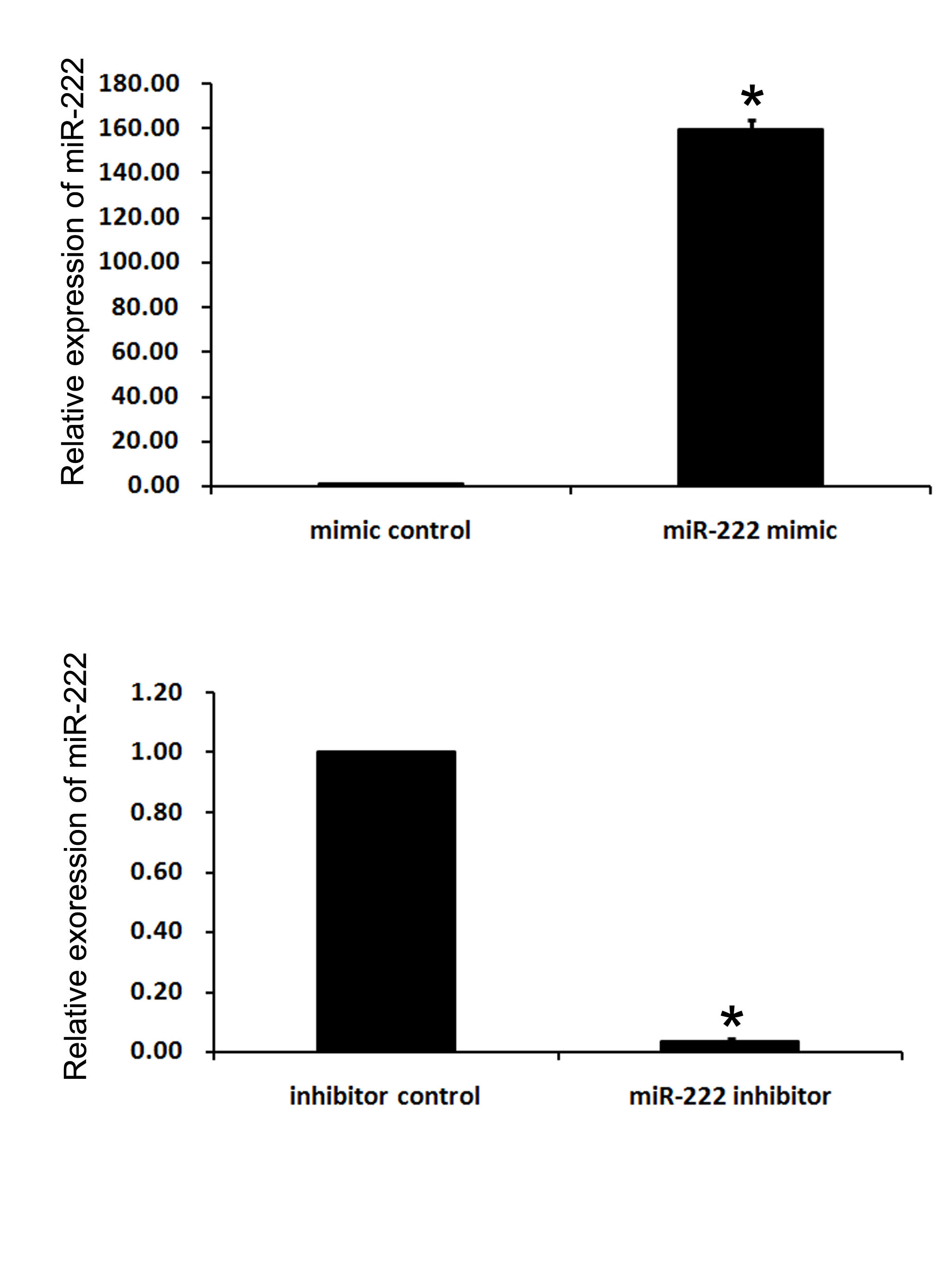

Supplement: Supplementary file 2 — Fig. S2. Transfection efficiency of miR‐222 mimics and inhibitors in SW480 cells as detected by RT‐PCR. Comparisons between groups were analyzed using t‐tests (two‐sided). Differences with P values of less than 0.05 are considered significant. [file FEB4-9-901-s002.tif]

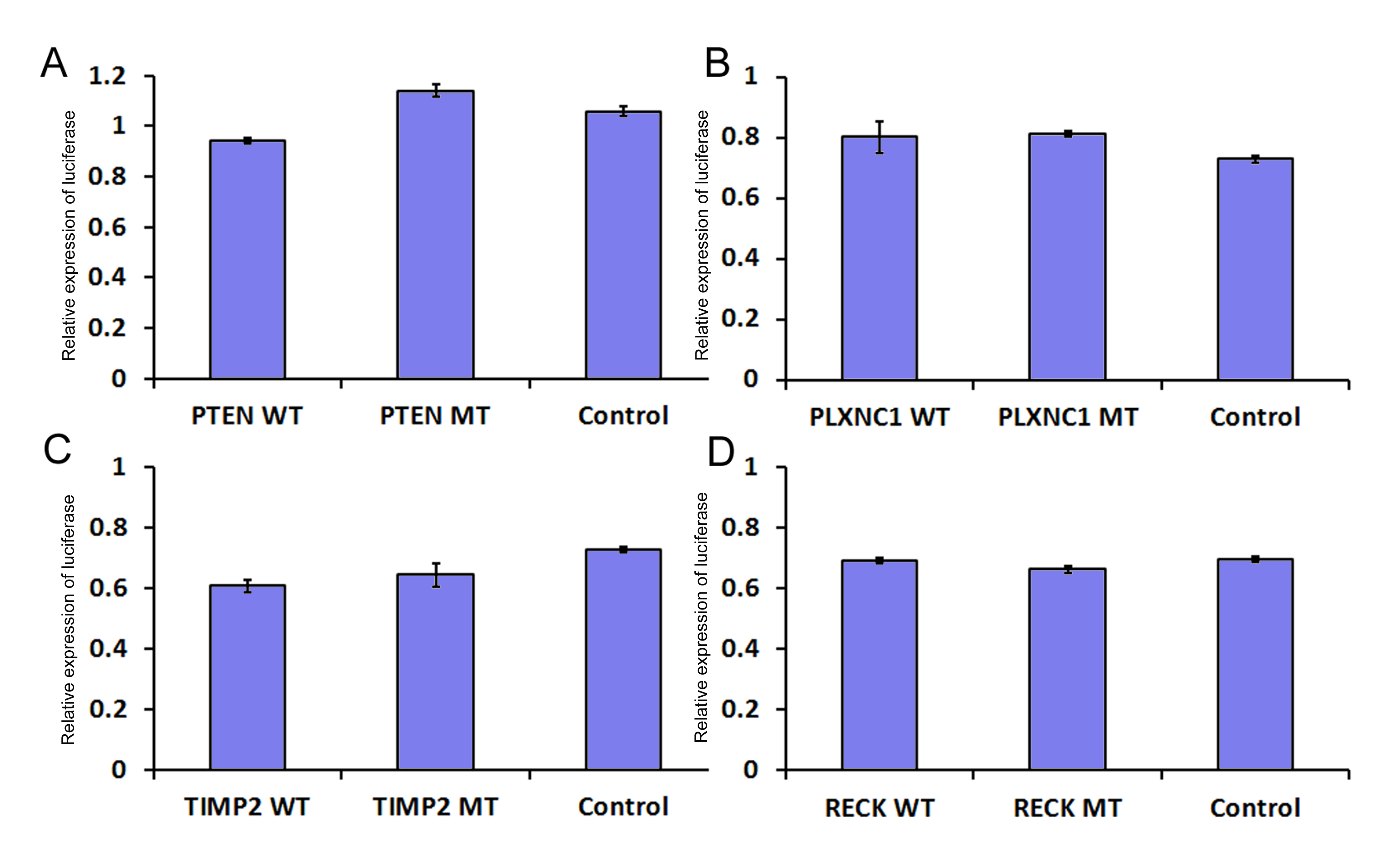

Supplement: Supplementary file 3 — Fig. S3. The luciferase activity after transfection of the indicated 3′‐UTR‐ or mutant 3′‐UTR‐driven reporter constructs for PTEN, PLXNC1, RECK and TIMP2. Comparisons between groups were analyzed using t‐tests (two‐sided). Differences with P values of less than 0.05 are considered significant. [file FEB4-9-901-s003.tif]

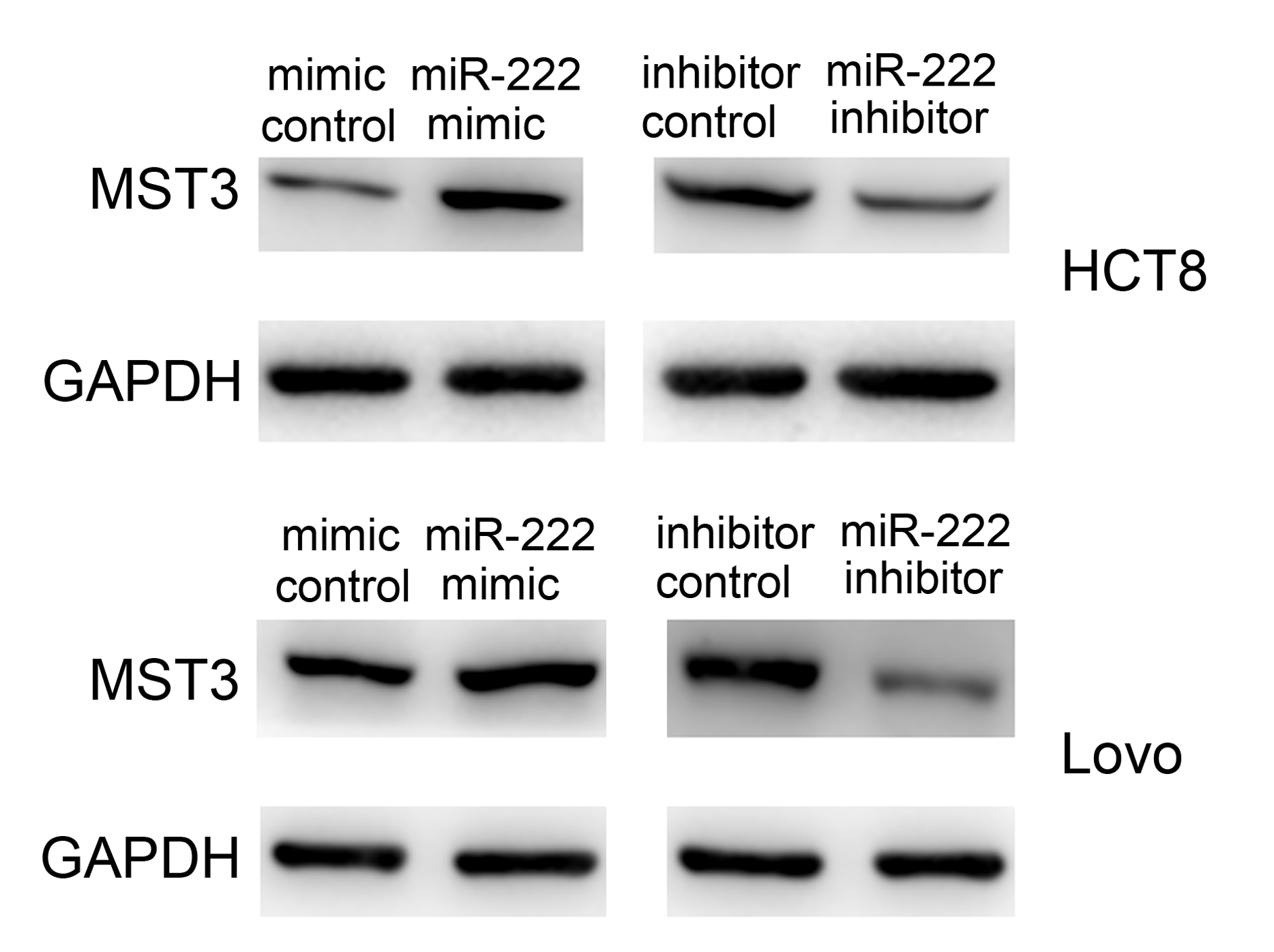

Supplement: Supplementary file 4 — Fig. S4. Western blot assay of MST3 protein levels in HCT8 and Lovo cells treated with miR‐222 mimics, mimics control, miR‐222 inhibitor and inhibitor control (n = 3). [file FEB4-9-901-s004.tif]

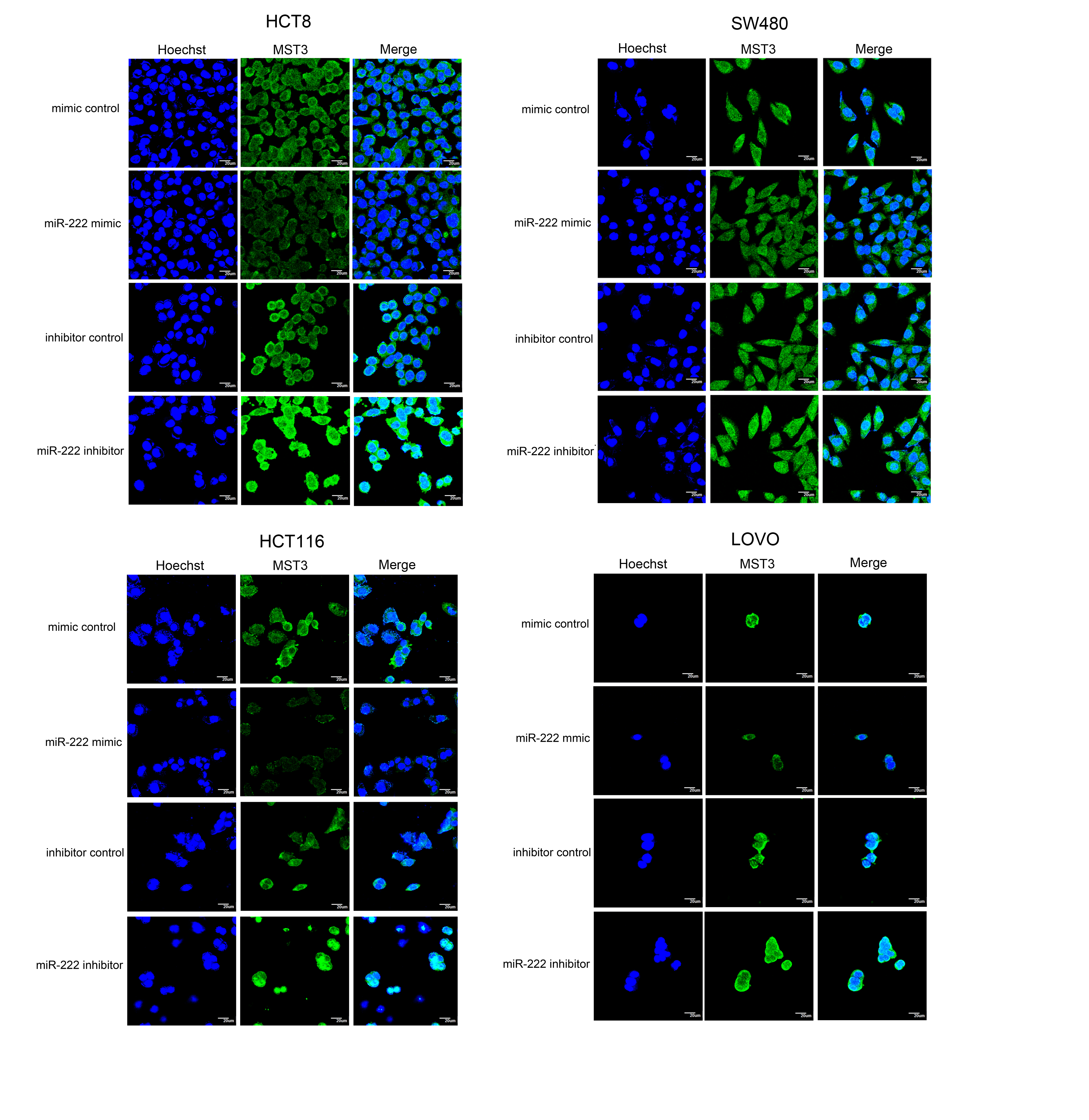

Supplement: Supplementary file 5 — Fig. S5. Immunofluorescence assay of miR‐222 on MST3 expression in CRC cell lines. The nucleus is blue (stained by Hoechst 33342) and MST3 is green (stained by Alexa Fluor® 488). Scale bar: 20 μm. [file FEB4-9-901-s005.tif]

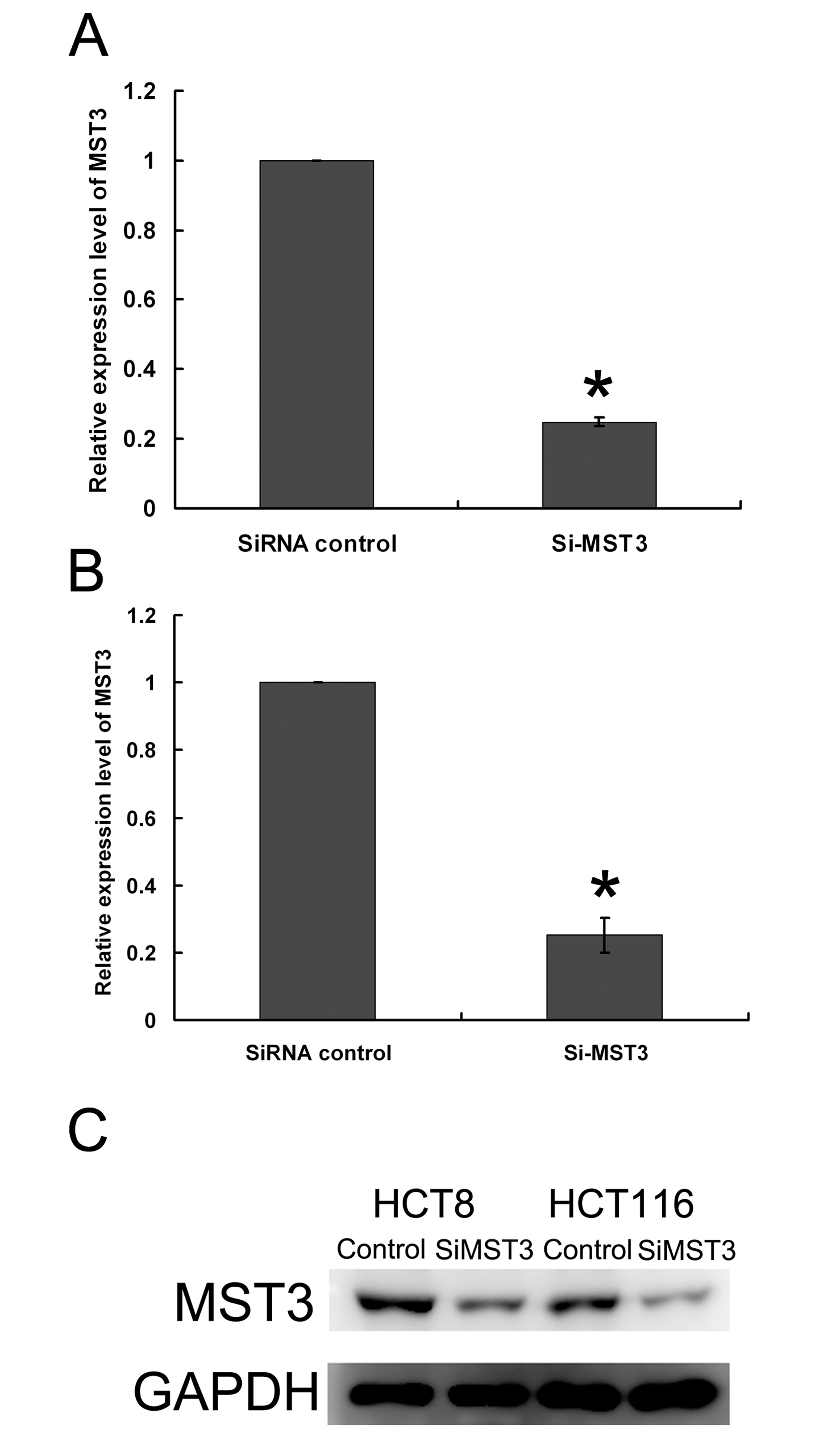

Supplement: Supplementary file 6 — Fig. S6. Interfering efficiency of MST3 siRNAs. (A,B) Interfering effects of MST3 siRNAs were detected by RT‐PCR. (A) HCT116 and (B) HCT8 cells (n = 3). Comparisons between groups were analyzed using t‐tests (two‐sided). Differences with P values of less than 0.05 are considered significant. (C) Western blot assay showed decreased MST3 expression after transfection with MST3‐siRNAs (200 nm). [file FEB4-9-901-s006.tif]

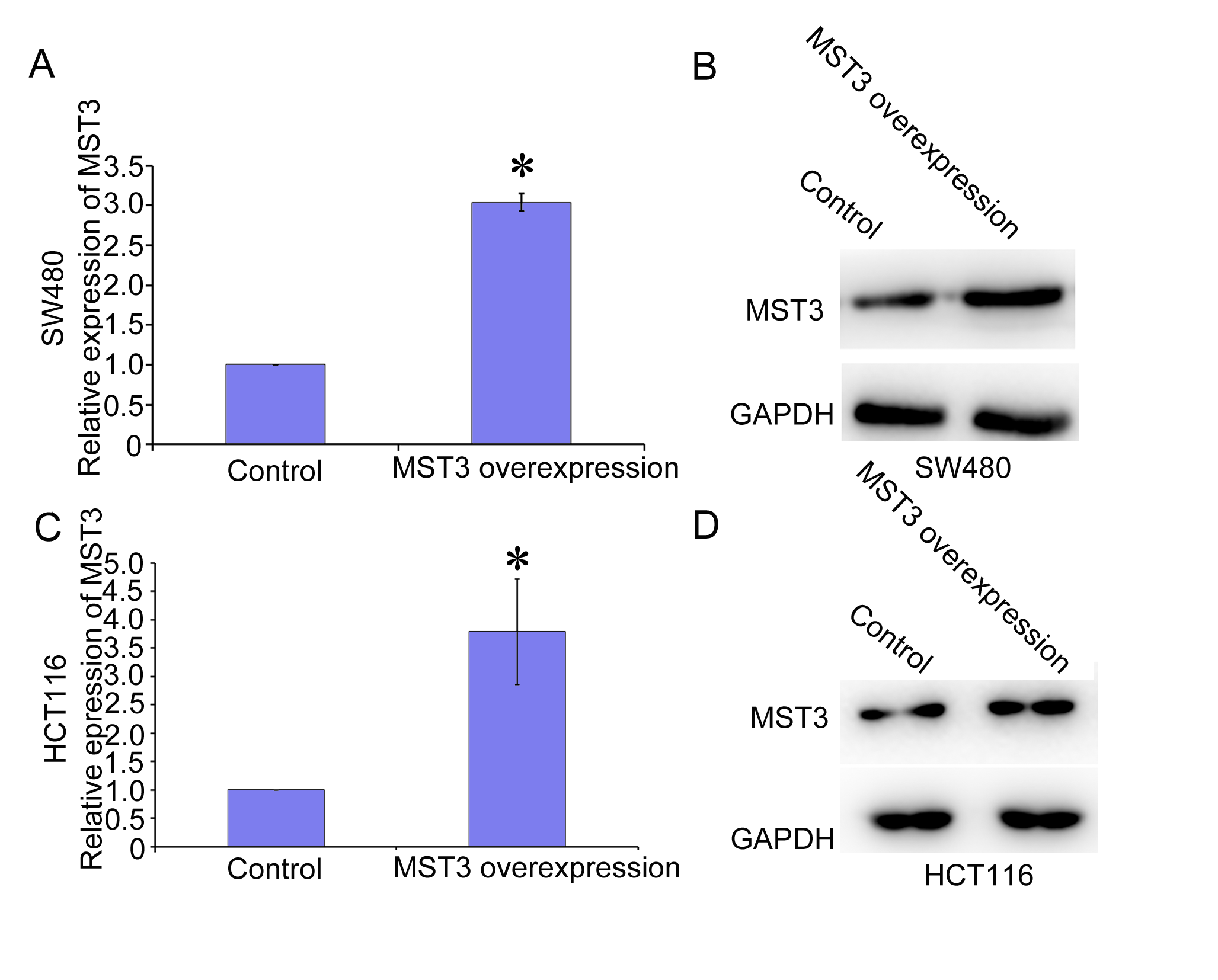

Supplement: Supplementary file 7 — Fig. S7. MST3 overexpression efficiency. MST3 expression was detected by RT‐PCR (A,C) (comparisons between groups were analyzed using t‐tests (two‐sided) and differences with P values of less than 0.05 are considered significant) and western blot (B,D) in SW480 (A,B) and HCT116 (C,D) cells. [file FEB4-9-901-s007.tif]

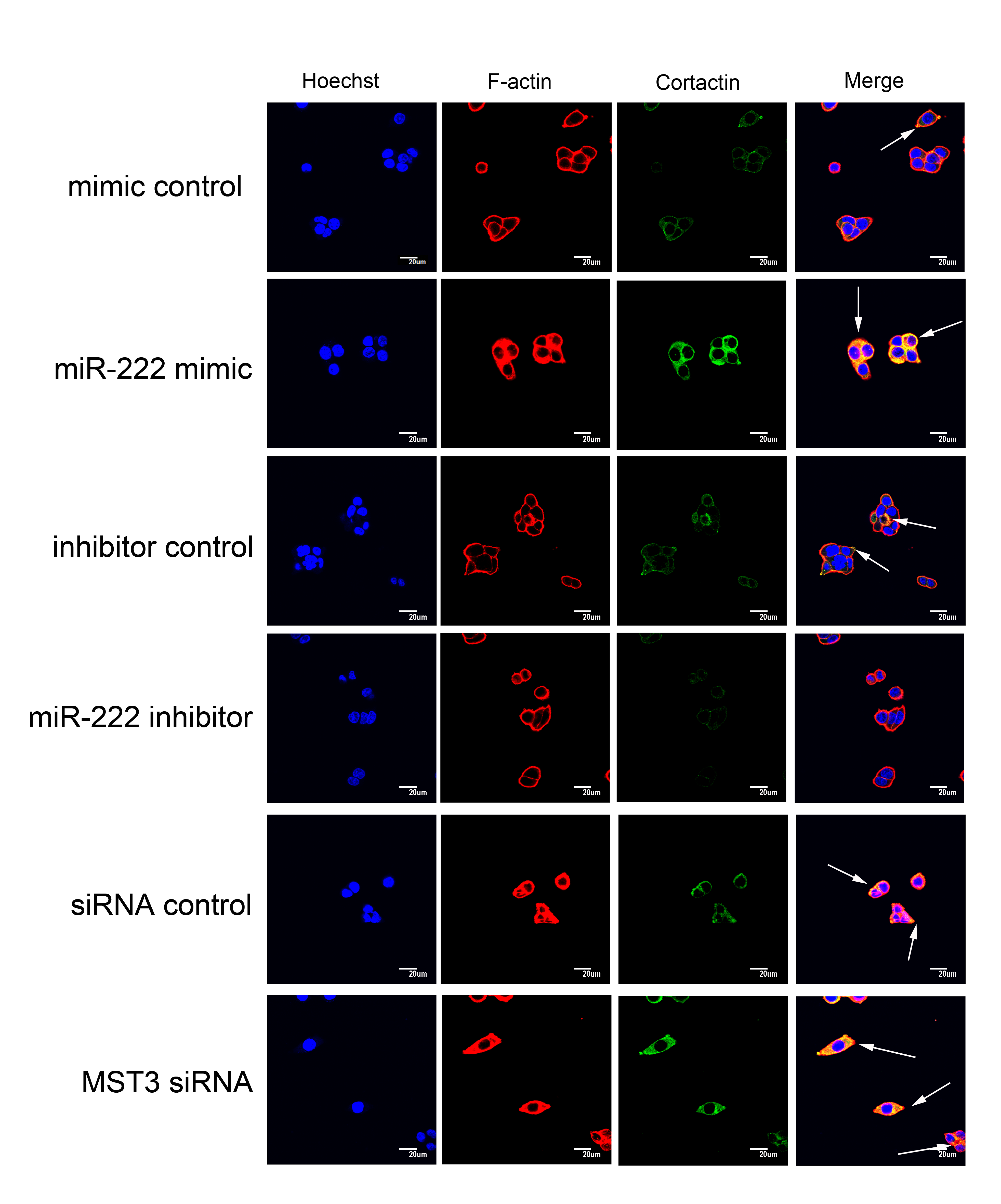

Supplement: Supplementary file 8 — Fig. S8. MiR‐222 and MST3 influence Lovo cell invadopodia formation. F‐actin (red) and cortactin (green) immunofluorescence images in Lovo cells with miR‐222 overexpression (mimics), miR‐222 inhibitor, MST3 interference and the respective controls. F‐actin‐ and cortactin‐positive puncta are indicative of invadopodia. The nucleus is blue (stained by Hoechst 33342). Scale bar: 20 μm. [file FEB4-9-901-s008.tif]

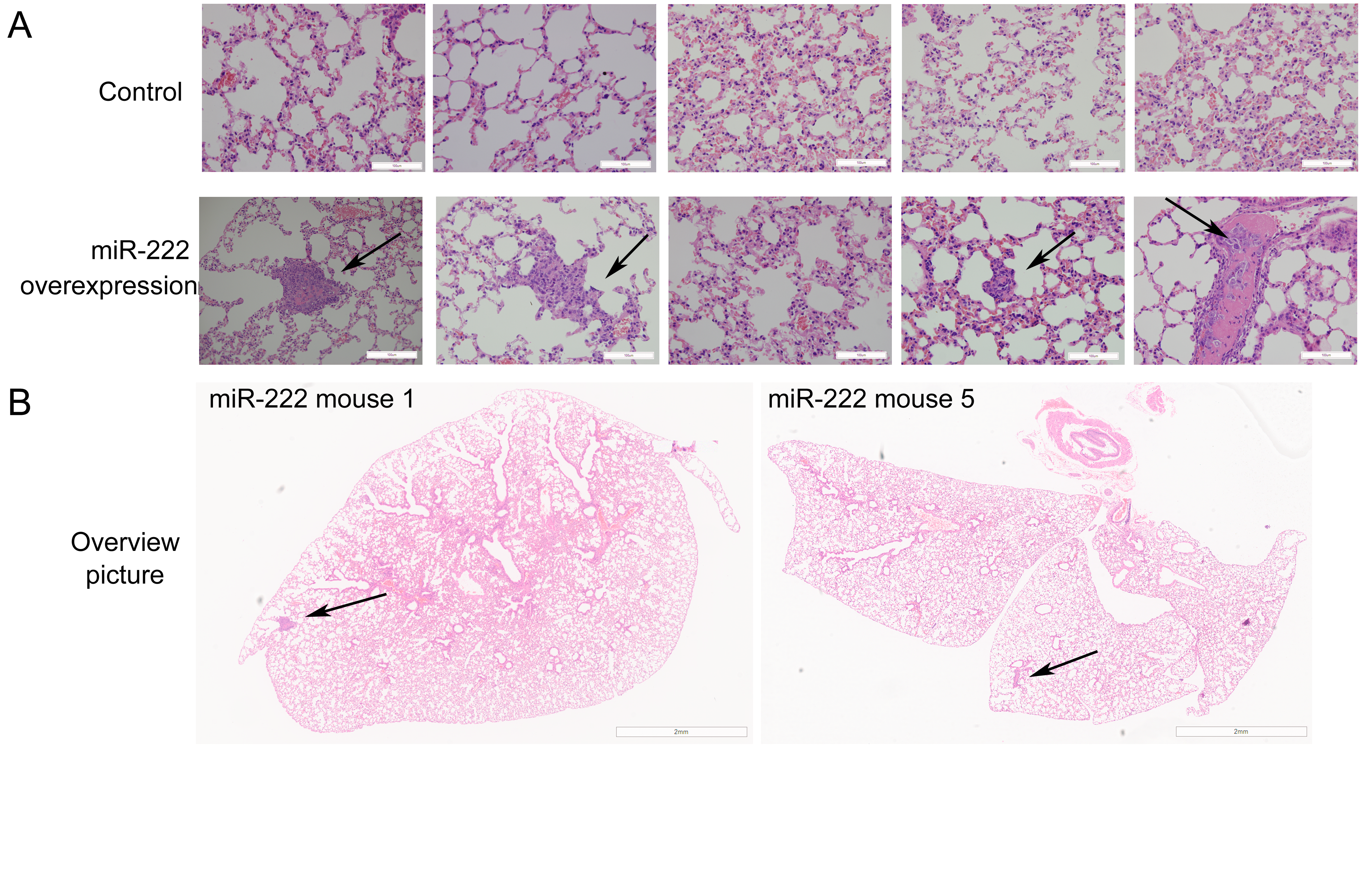

Supplement: Supplementary file 9 — Fig. S9. MiR‐222 promotes metastasis in mice. (A) Examination of lung tissues by hematoxylin‐eosin staining indicated no metastases in the HCT116‐NC inoculated mice (upper panel, 5/5) and metastases in the HCT116‐miR‐222 inoculated mice (lower panel, 4/5; the arrows indicate the metastatic sites). Scale bar: 100 μm. (B) Present overview picture of the lung sections in HCT116‐miR‐222 inoculated mice. Scale bar: 2 mm. [file FEB4-9-901-s009.tif]
