# Supplementary material for: microRNA‐222 promotes colorectal cancer cell migration and invasion by targeting MST3
Source: FEBS Open Bio. 2019 Apr 2;9(5):901–13. doi: 10.1002/2211-5463.12623 (PMC6487838; doi:10.1002/2211-5463.12623)
Supplement: Supplementary file 10 — Table S1. The primers (mRNA) for real time PCR. Table S2. The primers (miRNA) for real time PCR. Table S3. The sequence of target gene wild‐type and mutation of 3′‐UTR. Table S4. The correlation between miR‐222 and MST3 in 21 fresh liquid nitrogen‐frozen CRC cancer tissue from 2014 to 2015. Table S5. The expression miR‐222 and MST3 of colorectal cancer patients (2014–2015). [file FEB4-9-901-s010.docx]

Supple Table 1 The primer (mRNA) of real time PCR

| Gene | Forward primer(5'→3') | Reverse primer(5'→3') |
| --- | --- | --- |
| MST3 | AGGCATTGACAATCGGACTCA | CTGACTCAGCACTGTGATTTCT |
| E-Cadherin | CCCACCACGTACAAGGGTC | ATGCCATCGTTGTTCACTGGA |
| Vimentin | AGAACTTTGCCGTTGAAGCTG | CCAGAGGGAGTGAATCCAGATTA |
| ZO-1 | CAACATACAGTGACGCTTCACA | GACGTTTCCCCACTCTGAAAA |
| Fibronectin | CCCCATTCCAGGACACTTCTG | GCCCACGGTAACAACCTCTT |
| GAPDH | GGTCACCAGGGCTGCTTTTA | GAGGGATCTCGCTCCTGGA |

Supple Table 2 The primers (miRNA) of real time PCR

| miRNA | Primer Sequence(5'→3') |
| --- | --- |
| hsa-miR-222 | AGCTACATCTGGCTACTGGGT |
| U6 | CAAGGATGACACGCAAATTCG |

Supple Table3 The sequence of target gene wild tape and mutation of 3’UTR

| MST3  (Conserved) | WT | F | 5'TCGAGTTGCTCTTGTGAAGTGGCTGGTTTGGTTCTGTATGTAGCATTTTGTACCTTTCCTCTGGCAAAACACTGTCAGTTGC3' |
| --- | --- | --- | --- |
|  |  | R | 5'GGCCGCAACTGACAGTGTTTTGCCAGAGGAAAGGTACAAAATGCTACATACAGAACCAAACCAGCCACTTCACAAGAGCAA C3' |
|  | MT | F | 5'TCGAGTTGCTCTTGTGAAGTGGCTGGTTTGGTTCTGTTTTTGTACCTTTCCTCTGGCAAAACACTGTCAGTTGC3' |
|  |  | R | 5'GGCCGCAACTGACAGTGTTTTGCCAGAGGAAAGGTACAAAAACAGAACCAAACCAGCCACTTCACAAGAGCAA C3' |
| MST3  (poorly conserved) | WT | F | 5'TCGAGGAAGCAAGGAAGTTAGTGGGTGAGCTTGGGGATGTAGCTCAGCTATCTGCTGGTCTAGTGGCCTCTAAGCTATAGGGC3' |
|  |  | R | 5'GGCCGCCCTATAGCTTAGAGGCCACTAGACCAGCAGATAGCTGAGCTACATCCCCAAGCTCACCCACTAACTTCCTTGCTTC C3' |
|  | MT | F | 5'TCGAGGAAGCAAGGAAGTTAGTGGGTGAGCTTGGGGTCAGCTATCTGCTGGTCTAGTGGCCTCTAAGCTATAGGGC3' |
|  |  | R | 5'GGCCGCCCTATAGCTTAGAGGCCACTAGACCAGCAGATAGCTGACCCCAAGCTCACCCACTAACTTCCTTGCTTC C3' |
| PLXNC1 | WT | F | 5'TCGAGAAGTGGTTTTGTTTGAAAACTACAGCTATGTAGCACTTGTGCTACACTGCACCTCTGCATTGTAAAGGGATACTGCCGC3' |
|  |  | R | 5'GGCCGCGGCAGTATCCCTTTACAATGCAGAGGTGCAGTGTAGCACAAGTGCTACATAGCTGTAGTTTTCAAACAAAACCACTT C3' |
|  | MT | F | 5'TCGAGAAGTGGTTTTGTTTGAAAACTACAGCTCTTGTGCTACACTGCACCTCTGCATTGTAAAGGGATACTGCCGC3' |
|  |  | R | 5'GGCCGCGGCAGTATCCCTTTACAATGCAGAGGTGCAGTGTAGCACAAGAGCTGTAGTTTTCAAACAAAACCACTT C3' |
| PTEN | WT | F | 5'TCGAGTTTTGACACTTGTTGTCCAGTTGAAAAAAGGTTGTGTAGCTGTGTCATGTATATACCTTTTTGTGTCAAAAGGACAT GC3' |
|  |  | R | 5'GGCCGCATGTCCTTTTGACACAAAAAGGTATATACATGACACAGCTACACAACCTTTTTTCAACTGGACAACAAGTGTCAAAA C3' |
|  | MT | F | 5'TCGAGTTTTGACACTTGTTGTCCAGTTGAAAAAAGGTTGGTGTCATGTATATACCTTTTTGTGTCAAAAGGACAT GC3' |
|  |  | R | 5'GGCCGCATGTCCTTTTGACACAAAAAGGTATATACATGACACCAACCTTTTTTCAACTGGACAACAAGTGTCAAAA C3' |
| TIMP2 | WT | F | 5'TCGAGGCAGAAACTTTTGAGGGTCGTTGCAAGACTGTGTAGCAGGCCTACCAGGTCCCTTTCATCTTGAGAGGGAGC3' |
|  |  | R | 5'GGCCGCTCCCTCTCAAGATGAAAGGGACCTGGTAGGCCTGCTACACAGTCTTGCAACGACCCTCAAAAGTTTCTGC C3' |
|  | MT | F | 5'TCGAGGCAGAAACTTTTGAGGGTCGTTGCAAGACTGGGCCTACCAGGTCCCTTTCATCTTGAGAGGGAGC3' |
|  |  | R | 5'GGCCGCTCCCTCTCAAGATGAAAGGGACCTGGTAGGCCCAGTCTTGCAACGACCCTCAAAAGTTTCTGC C3' |
| RECK | WT | F | 5'TCGAGCACTTAAAGCTTTCAGAATATGTCAGTGCTGATGTAGCATGCTTGTTGCAATTGCCTTTTTTCTGTATA GC3' |
|  |  | R | 5'GGCCGCTATACAGAAAAAAGGCAATTGCAACAAGCATGCTACATCAGCACTGACATATTCTGAAAGCTTTAAGTG C3' |
|  | MT | F | 5'TCGAGCACTTAAAGCTTTCAGAATATGTCAGTGCTGGCTTGTTGCAATTGCCTTTTTTCTGTATA GC3' |
|  |  | R | 5'GGCCGCTATACAGAAAAAAGGCAATTGCAACAAGCCAGCACTGACATATTCTGAAAGCTTTAAGTG C3' |

Supple Table 4 The correlation between miR-222 and MST3 in 21 fresh liquid nitrogen-frozen CRC cancer tissue from 2014-2015. Correlations between miR-222 and MST3 expression were determined with the Pearson assay and correlation with the bivariate Kendall’s tau-b assay.


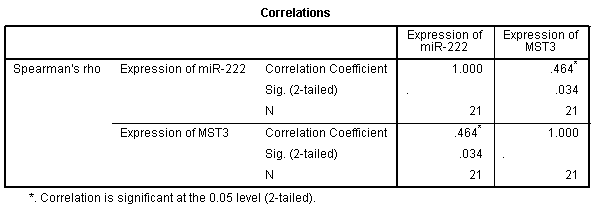


Supple Table5The expression miR-222 and MST3 of colorectal cancer patients (2014-2015)

| Number | Site | Histology | Histological grading | Stage | Expression of miR-222  （ΔΔCT） | Expression of MST3 |
| --- | --- | --- | --- | --- | --- | --- |
| 1 | colon | Adenocarcinoma | Middle | IIIB | 5.91 | ++ |
| 2 | colon | Adenocarcinoma | Middle | IIIB | 6.55 | + |
| 3 | colon | Adenocarcinoma | Middle | IIIB | 2.48 | - |
| 4 | colon | Adenocarcinoma | Middle | IIIB | 4.39 | + |
| 5 | colon | Adenocarcinoma | Middle | IIIC | 3.04 | + |
| 6 | colon | Adenocarcinoma | Middle | IIIB | 4.27 | + |
| 7 | colon | Adenocarcinoma | Middle | IIIB | 6.01 | + |
| 8 | colon | Adenocarcinoma | Middle-low | IIIB | 4.99 | + |
| 9 | colon | Adenocarcinoma | High-middle | IIIB | 5.24 | + |
| 10 | colon | Adenocarcinoma | Middle | IIIB | 4.97 | + |
| 11 | colon | Adenocarcinoma | Middle-low | IIIB | 5.08 | ++ |
| 12 | colon | Adenocarcinoma | Middle | IIIB | 1.67 | - |
| 13 | colon | Adenocarcinoma | High-middle | IIIB | 4.36 | - |
| 14 | colon | Adenocarcinoma | Low | IIIB | 6.55 | ++ |
| 15 | rectum | Adenocarcinoma | Middle | IIIB | 5.14 | ++ |
| 16 | rectum | Adenocarcinoma | Middle | IIIB | 5.17 | + |
| 17 | rectum | Adenocarcinoma | Middle | IIIB | 5.26 | ++ |
| 18 | rectum | Adenocarcinoma | Middle | IIIB | 4.6 | + |
| 19 | rectum | Adenocarcinoma | Middle | IIIB | 5.28 | ++ |
| 20 | rectum | Adenocarcinoma | Middle-low | IIIC | 4.53 | - |
| 21 | rectum | Adenocarcinoma | Middle | IIIA | 5.68 | ++ |
